# Tubal Pregnancy—Its Diagnosis and Treatment
1A paper read before the South-Western Branch of the British Medical Association, March 6th, 1906.


**Published:** 1906-06

**Authors:** C. Hamilton Whiteford


### TUBAL PREGNANCY—ITS DIAGNOSIS AND TREATMENT.[1]

BY

C. Hamilton Whiteford, M.R.C.S., L.R.C.P.

Cases of tubal pregnancy are not very rare. During the past fourteen years I have had opportunities, while attending hospital practice, of seeing twenty examples, in addition to the three cases occurring in my private work. My remarks will be limited to tubal pregnancy during its early stages,

[1] A paper read before the South-Western Branch of the British Medical Association, March 6th, 1906.

the large majority of cases coming under the care of the medical man during the first few months of their existence. I also wish to point out that the medical man is usually sent for only after rupture of, or hæmorrhage and abortion from, the gravid tube has already commenced.

A woman of child-bearing age, frequently a *primipara*, or if a *multipara* with an interval of several years since her last pregnancy, is seized with severe pain in the lower abdomen, followed by faintness, sickness, and vaginal loss of blood. There has been a missed or a delayed period. The patient and her friends assume that a miscarriage is in progress, and the patient retires to bed. Then the illness may take one of the two following forms:—

(*a*) With rest in bed the patient temporarily improves, the pain and vaginal hæmorrhage diminish or disappear, only however to return, especially if the patient tries to leave her bed. This alternate cessation and return of pain and hæmorrhage continues, and at length, one to three or more weeks from the onset of the illness, the medical man is called in. Fortunately for everyone concerned, this remittent form of the illness is by far the more common.

(*b*) The less common, but the more dangerous form is that in which the first attack of pain and vaginal hæmorrhage is quickly followed by severe and increasing collapse, with a rapid, gradually vanishing pulse, a subnormal temperature, repeated faintings, and blanching of the whole body. The medical man, arriving a few hours after the first attack of pain, finds the patient blanched and almost pulseless.

Taking in detail a few of the prominent symptoms:—

*Amenorrhœa.*—There is frequently a history of a missed or a delayed period, but it is not uncommon to find that the last period, although commencing at the expected time, instead of lasting a few days, has continued almost incessantly for some weeks. Another variety of menstrual irregularity is that in which the normal period took place only a week or two before the attack of pain and bleeding.

*Pain.*—This is felt in the lower abdomen, and is sudden and acute, often causing faintness and sickness. The patient can usually differentiate one side of the abdomen as being more painful than the other. When the pain subsides, tenderness of the lower abdomen and pelvic contents remains. The pain is apt to return on a recurrence of the hemorrhage.

*Hæmorrhage.*—Bleeding from the vagina is usual but not invariable. The blood differs from menstrual blood in being darker and in containing clots. The flow may continue intermittently for weeks, sometimes a membranous cast of the uterus is passed, but as a rule the uterine decidua disintegrates and comes away as dêbris. Discovery of chorionic villi in any piece of membrane passed is almost diagnostic of an intra-uterine pregnancy. The blood lost *per vaginam* in tubal pregnancy comes partly from the uterus and partly from the pregnant tube, which not only bleeds into the pelvis but also into the portion of the tube lying between the pregnancy and the uterus.

*Temperature.*—In the fulminating type the temperature is subnormal. In the remittent type, in which a mass of blood is formed in the pelvis, the temperature is usually raised. This pyrexia was, until recently, attributed to the formation of a ferment in the coagulated blood, but Dudgeon and Sargent,[1] in examining seventeen consecutive cases of ruptured tubal pregnancy, found in the extravasated blood of every case a white staphylo-coccus.

*Diagnosis.*—In a case of ruptured tubal pregnancy successfully treated by operation, nine-tenths of the credit belongs to the diagnostician, the operator's task is comparatively easy. In all cases, prior to examination, the bladder must be emptied by catheter. In cases where tenderness or rigidity of the abdomen renders bimanual examination unsatisfactory, an anæsthetic should be employed. Examination *per rectum* is often helpful. I would suggest as a preliminary axiom that "in the absence of symptoms of

[1] *Lancet*, 1905, i. 473.

severe internal hæmorrhage, and in the absence of a pelvic tumour, which can be differentiated from the uterus, a diagnosis of ruptured tubal pregnancy is unjustifiable."

*The Fulminating Type of Rupture.*—In this the surgeon has little beyond the symptoms and signs of severe persistent internal bleeding on which to base his diagnosis. There is, perhaps, a history of a delayed or missed period, followed by sudden acute pain in the lower abdomen, with a vaginal loss of blood. There is no definite pelvic or abdominal tumour to be felt. The onset may, at first, suggest a perforation of one of the hollow viscera, but perforation of a hollow viscus is rarely accompanied by vaginal hæmorrhage, and the events of the next few hours—the progressive anæmia, the rapid and failing pulse, with persistent subnormal temperature—all point to severe and progressive loss of blood out of all proportion to the amount lost *per vaginam.* Nothing but severe internal bleeding will explain these symptoms, and with a history such as the above the usual source of such hæmorrhage is a pregnant tube.

*The Remittent Type of Rupture.*—Here the patient has been laid up for many days or weeks. The pelvis and sometimes the lower abdomen is occupied by a boggy tumour, the tenderness of which varies in individual cases. In a tubal pregnancy, the enlarged uterine vessels can often be felt pulsating in the vaginal vault on the affected side. The key to the situation is the exact determination of the position of the uterine fundus, and the condition of its cavity. As Taylor expresses it: "The exact position of the fundus and the emptiness or non-pregnant state of its cavity are very important and sometimes very difficult points to determine, but they must be decisively settled before any operation is undertaken." In any case in which symptoms are not urgent and in which the examination is inconclusive it will be found a good plan to simply await developments. At the end of a week the local conditions may have so altered that a correct diagnosis becomes comparatively easy. Only after all other means have failed should the uterine sound be passed.

*Differential diagnoses* have to be made from:—

1. *Simple Abortion.*—In this no tumour can be demonstrated apart from the fundus uteri, which is enlarged. If bleeding persists, explore the interior of the uterus, and thereby decide the questions of—
   (*a*) Miscarriage of intrauterine pregnancy.
   (*b*) Fibroid.
   (*c*) Cancer.
2. *Retroverted Gravid Uterus.*—Taylor's axiom is, "Never to diagnose a gravid retroflexion without careful exclusion of extra-uterine pregnancy"; but the converse appears even more important, namely, "Never to diagnose a tubal pregnancy without first ascertaining that the mass felt is something more than an enlarged fundus uteri." Here the bimanual examination under an anæsthetic demonstrates that the mass felt in Douglas's pouch is continuous with the cervix, and that there is in the pelvis no other tumour which could be the fundus.
3. *Intrauterine Pregnancy, complicated with Pelvic Tumour.*—Demonstrate the position of the fundus in relation to the tumour, also ascertain if the uterus is enlarged. Defer the passage of a sound to the latest possible date. Where symptoms are not urgent watch the case, and observe whether it is the uterus or the tumour which is increasing in size. Assuming the presence of a slightly enlarged uterus with a tender pelvic swelling in a patient who has had amenorrhœa, or a delayed period, followed by sudden abdominal pains with vaginal hæmorrhage, what are the probabilities? The extreme probability is that the mass is composed of blood-clot, tube, ovary, adherent omentum and bowel, the result of rupture or abortion of a pregnant tube. The possibility is that the pregnancy may be in the uterus, and the pelvic mass may be—
   (*a*) An inflamed tube or ovary.
   (*b*) An ovarian cyst either

1. Suppurating; or
2. Full of blood and adherent to surrounding structures owing to a twisted pedicle.

(*c*) An appendix abscess.

(*d*) A subperitoneal myoma, which is rarely the cause of pelvic pain unless its blood supply is interfered with, which might occur if it had a narrow pedicle and this pedicle became twisted.

Assuming that, after a period of expectant treatment, a diagnosis of rupture of a pregnant tube is made, and on operation being performed one of the above-mentioned conditions is discovered little harm has been done. There has been an error in diagnosis but not in treatment, since the safest treatment for diseased appendages, ovarian cyst, myoma with twisted pedicle or appendix abscess, is their removal by operation, even supposing that the uterus is gravid. It is remarkable how many cases of pregnancy, complicated by the presence of a pelvic tumour, proceed after removal of this tumour uninterruptedly to term.

*Treatment*:—

(*a*) *In the Fulminating Type*.—The only chance for the patient lies in immediate operation. This must be performed at the patient's house. It is absolutely unjustifiable to move to a hospital or nursing home a patient who is nearly dead from loss of blood.

(*b*) *In the Remittent Type*.—Small pelvic extravasations of blood are frequently absorbed without causing symptoms. Therefore, given a patient with a normal temperature and a small mass of blood which is not tender, and is week by week diminishing in size, expectant treatment is justifiable. But so long as any portion of the blood-clot remains unabsorbed there must always exist the risk of infection from neighbouring bowel, in which event operation would become imperative. While conceding the justifiability of expectant treatment in cases such as the above, after duly weighing every case which has come under my notice, I am forced to the conclusion that the safest, and therefore the best, surgery is to remove in every case the extravasated blood and the damaged tube.

---

### THE NEW THEORY AND PROPHYLAXIS OF PUERPERAL ECLAMPSIA.[1]

BY

ELIZA L. WALKER DUNBAR, M.D.

